# Effective Evaluation of Medical Images Using Artificial Intelligence Techniques

**DOI:** 10.1155/2022/8419308

**Published:** 2022-08-10

**Authors:** S. Kannan, G. Premalatha, M. Jamuna Rani, D. Jayakumar, P. Senthil, S. Palanivelrajan, S. Devi, Kibebe Sahile

**Affiliations:** ^1^Department of Electronics and Communication Engineering, Mallareddy Institute of Technology and Science, Secunderabad 500100, Telangana, India; ^2^Department of Computer Science and Engineering, Mohamed Sathak A. J College of Engineering, Sipcot IT Park, Siruseri, Chennai 603103, Tamilnadu, India; ^3^Department of Electronics and Communication Engineering, Sona College of Technology, Salem 636005, Tamilnadu, India; ^4^Department of Electronics and Communication Engineering, Kuppam Engineering College, Kuppam 517425, Andhra Pradesh, India; ^5^Department of Electronics and Communication Engineering, SRM Institute of Science & Technology, Ramapuram, Chennai 603203, Tamilnadu, India; ^6^Department of Electronics and Communication Engineering, M. Kumarasamy College of Engineering, Thalavapalayam, Karur 639113, Tamilnadu, India; ^7^Department of Electronics and Communication Engineering, Sri Venkateswara College of Engineering, Nellore, Andhra Pradesh-52316, India; ^8^Department of Chemical Engineering, College of Biological and Chemical Engineering, Addis Ababa Science and Technology University, Addis Ababa, Ethiopia

## Abstract

This work is implemented for the management of patients with epilepsy, and methods based on electroencephalography (EEG) analysis have been proposed for the timely prediction of its occurrence. The proposed system is used for crisis detection and prediction system; it is useful for both patients and medical staff to know their status easily and more accurately. In the treatment of Parkinson's disease, the affected patients with Parkinson's disease can assess the prognostic risk factors, and the symptoms are evaluated to predict rapid progression in the early stages after diagnosis. The presented seizure prediction system introduces deep learning algorithms into EEG score analysis. This proposed work long short-term memory (LSTM) network model is mainly implemented for the identification and classification of qualitative patterns in the EEG of patients. While compared with other techniques like deep learning models such as convolutional neural networks (CNNs) and traditional machine learning algorithms, the proposed LSTM model plays a significant role in predicting impending crises over 4 different qualifying intervals from 10 minutes to 1.5 hours with very few wrong predictions.

## 1. Introduction

Seizure prediction methodologies are based entirely on continuous EEG recordings. Unlike seizure detection, the sample's weight now shifts to epileptic waves at EEG intervals before seizure onset. Qualified EEG analysis for seizure prediction has two main approaches. The first is based on the analysis of the characteristics of export signals to track the temporal change in their prices, which leads to the onset of a crisis. When prices exceed a certain threshold, the system is activated to warn of an impending crisis. From the time the alert is issued, a window of time is given during which a crisis is expected to eventually occur. If the crisis occurs within the forecast window, it is considered to have been successfully forecast; otherwise, it is characterized as an incorrect forecast. The second approach is based on the use of machine learning to identify subcritical and intra-critical areas of the EEG of patients. In this case, the scoring window and the corresponding EEG segments from the start point of each attack to the window definition are determined before grouping them in the same class as the markers. All other sections of the EEG preceding the scoring window and all sections after the end of the seizure are intercritical. After exporting two classes, the classification algorithm learns to separate them. Each time, a part of the EEG is deemed qualified; a corresponding warning of an impending crisis is generated. The qualifying window in both approaches is randomly chosen by investigators in each study and has been shown to last from minutes to hours before a seizure.

## 2. Literature Review

In recent years, machine learning algorithms have been widely used to predict crises. In said system, a model was developed and trained for the classification of the rating and average critical departments using support vector machines (SVMs) using the characteristics of spectrum extraction for each EEG channel and energy distribution in different frequency ranges [1]. Similar analysis methods have shown that the EEG frequency composition changes significantly over the qualifying period. In their study, Netoff and his colleagues used EEG energy distribution in 9 frequency bands (0.5 to 4 Hz, 4 to 8 Hz, 8 to 13 Hz, 13 to 30 Hz, 30 to 50 Hz, 50 to 70 Hz, 70–90 Hz, and > 90 Hz) and an SVM classifier to separate the qualifying and mesocritical segments. The evaluation was performed on the EEG recordings of 9 patients at the Freiburg base (45 seizures in 219 hours) with a mean sensitivity of 77.8% without prediction errors [2]. The qualifying window was much shorter at 5 minutes. In a similar study evaluating a large sample of 18 patients from Freiburg (80 seizures in 433 hours), the researchers focused on extracting features from the higher frequency spectrum and the methodology achieved a significantly higher average sensitivity of 97.5%, but also 0.27 predictions per hour [3]. And in this study, the classification was performed using SVM. In addition to the Fourier transform and the corresponding wavelet transform, it has also been shown to be a very efficient method of calculating the energy distribution of the signal to separate the defining parts of the EEG both intracranial [4] and on the surface recordings [5] with similar results. In another approach, the number of null transitions (sign change in the EEG waveform) was used to determine qualifying sites and predict seizures. In the proposed methodology, the differences in the rate of zero transitions between qualified and intercritical units were studied using Gaussian mixed models (GMMs) to predict 40-minute depth crises [6]. To improve neural networks, models have been proposed that are capable of synthesizing more efficient complex networks that can be better adapted to train data and learn more complex representations and hidden dependencies. In recent years, deep learning algorithms have become increasingly popular in medical image and signal analysis due to the increase in available computing power and the collection of large amounts of data [7]. One of the best-known deep learning models is convolutional neural networks (CNNs), a type of network consisting of repeated layers of convolution and pooling that is very efficient for analyzing data representing a lattice topology, such as medical images [8]. However, some studies have also been suggested, presented to CNN on EEG analysis for the purpose of predicting seizures. One of these methodologies used a CNN network with 3 successive hidden convergence levels to estimate the spectrum of EEG signals extracted using the short-term Fourier transform. The spectrum was analyzed as an image to find differences between qualifying and mesocritical segments [9]. Training of the deeper CNN with 6 hidden layers and use of the wavelet transform to extract frequency information from EEG [10].

## 3. Crisis Prediction Model with Deep Machine Learning Algorithms

**Figure 1:**
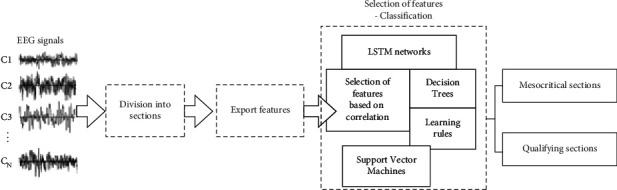
The basic stages of seizure prediction methodology.

This section presents a proposed methodology for predicting seizures from EEG data. The proposed methodology is based on the separation of the qualified EEG regions from the corresponding mesocritics using traditional classification algorithms [11], as well as deep learning models [12]. The process of EEG signal analysis and feature extraction for classifier training is carried out separately for each patient. The main stages of the technique are shown in Figure 1. In the first stage of data preprocessing, an initial evaluation of the available EEG recordings is performed to determine the number of channels available and the recording model when receiving signals. This is necessary because both the channels and the recording schema contained in the patient's charts may change during a long continuous EEG recording. By collecting information relevant to each patient's record file, channels that are not available in the entire record are discarded in subsequent analysis to ensure that the system consistently obtains the same amount of information from the patient's EEG, regardless of the log point parsed at any time.

Apart from checking the homogeneity of the EEG channels, no other preprocessing is applied to the data (e.g., filtering to exclude static noise or possible spurious endogenous or extracerebral parameters). The next step consists of dividing the EEG signals into segments of shorter duration, which will be analyzed separately to extract characteristics from which the final classification will be made. The duration of these sections is set to 5 seconds, and consecutive sections are separated without overlapping. The most well-known EEG processing and analysis methods are used to extract features. The set of exported features contains values from the EEG analysis in the time field and in the frequency field, from the calculation of the correlation between its different channels and from the trace, in an attempt to create a space of features that contains the complete information as possible. The advantage of using a feature extraction step is that it is an efficient method for revealing hidden and more complex correlations that may be hidden in the EEG signals. Next, the effect of feature extraction on the efficiency of the final classification is estimated in comparison with the direct use of the signals of each EEG channel in the form of a time series.

The last stage consists of training the classifier, which is in charge of dividing the sections into classificatory and mesocritical, using the values of the exported characteristics. The accuracy of crisis forecasting depends largely on the classification algorithm; therefore, in the following blocks different methods are evaluated, in the field of both classical machine learning (RIPPER algorithm, decision trees, SVM) and deep learning (LSTM model). Initially, each 5-second section is assigned to the appropriate class to which it belongs (critical, intercritical, or qualifying) based on the length of the qualifying window and the start and end points of each crisis, as specified in the database of data scores. File judgment segments are automatically discarded as they have no useful value for prediction, leading to a binary classification problem with two classes (mesocritic qualifier). The length of the qualifying period is an arbitrary choice for any study, as it has not yet been proven that there is a strictly defined period of time before the onset of each crisis. Several studies have even shown that changes in EEG activity can occur several hours before the onset of the disease [13–15]. For the sake of completeness, this article estimates 4 qualifying windows ranging from 15 minutes to 2 hours before the onset of crises.

## 4. Export Features

Features are extracted separately for each part of the EEG by analyzing the signal values of all available channels. The number of samples in each 5-second segment depends on the sample rate selected when the data were written (e.g., 1280 samples for 256 Hz). Exported features are among the most widely used in EEG analysis, and previous studies have also shown them to be very useful for seizure prediction, as their values change significantly over the scoring period.

### 4.1. Features in the Field of Time

This category includes features that can be calculated directly from recorded EEG samples at the time they are acquired. These features include average price (mean), variance, standard deviation, skewness, kurtosis, number of zero crossings, signal width, signal-to-peak (V-peak), and signal area with a trapezoid rule. All functions are exported separately for each EEG channel. Although the above measurements are relatively simple, they have great potential for detecting qualitative changes in patients' EEGs. For example, variability has been shown to decrease significantly during the rating period, while curvature increases as the onset of crisis approaches [16]. Also, as mentioned above, the number of spikes and zero crossings varies considerably over the rating period.

### 4.2. Characteristics in the Frequency Domain

EEG frequency analysis is one of the most useful methods, since the distribution of the signal energy hides a lot of information about the state of the brain. Therefore, the distribution of the signal energy in the main EEG frequencies is calculated: *δ* (1–3 Hz), *θ* (4–7 Hz), *α* (8–13 Hz), *β* (14–30 Hz), *γ*^1^ (31–55 Hz), and *γ*^2^ (65–110 Hz). The EEG spectrum for energy distribution is exported as a periodic table (periodogram, P) based on discrete Fourier transform (DFT). Also, in addition to the power distribution over the above 6 speeds, the total signal power of each channel is used.

**Figure 2:**
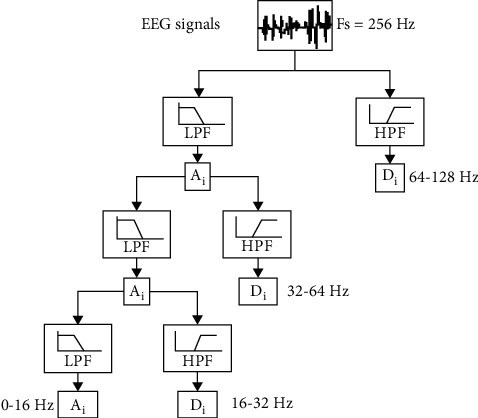
Illustration of the discrete 3-level transformation with wavelets.

A discrete wavelet transform (DWT) is then applied to the EEG signals of each section to calculate the energy distribution using the pyramidal algorithm proposed by [17], with the key feature of low computational cost. The transformation occurs in successive steps in an iterative process that each time separates the information contained in the high-frequency signal values by applying high-pass and low-pass composite filters to the original wavelet. In the present analysis, the fourth Daubechies wavelet was chosen as the initial wavelet. At each level, the signal samples are halved due to filtering. The procedure is tentatively illustrated in Figure 2 for 3 levels at a sample rate of 256 Hz, which is the CHB-MIT base sample rate that will be used to evaluate the methodology. The coefficients Di are called detail coefficients, while the approximation coefficients Ai are used to determine the outputs of the high-pass and low-pass filters, respectively. With the help of DWT, it is possible to estimate the energy distribution of the signal at individual sub-frequencies 64–128 Hz, 32–64 Hz, 16–32 Hz, 8–16 Hz, 4–8 Hz, 2–4 Hz, 1-2 Hz, and <1 Hz with low computational cost.

The use of 7 levels allows to calculate the energy distribution in the spectra very close to the base frequencies, adequately extracting the *δ* frequency at 1–3 Hz and discarding frequencies <1 Hz, which usually contain strong spurious potentials.

### 4.3. Channel Correlation and Self-Correlation

Calculating the correlation between different EEG channels (cross-correlation) can provide information on the simultaneous activation of different brain regions, since it has been shown that both synchronization and de-synchronization between them can indicate an impending crisis. The correlation is tested in pairs between all the possible combinations of available channels and is calculated based on the act of convergence (*∗*) from the following equation, taking into account the temporal offset *n* between the signals (delay time or delay):

(1) $$\begin{array}{c} \rho_{c_{i}c_{j}\left[m+n\right]=\left(\left(\left(c_{i}*c_{j}\right)\left[m+n\right.\right)/\sigma_{c_{i}}\sigma_{c_{j}}\right),\;n\in \left[-m,+m\right]}. \end{array}$$

To calculate the correlation coefficient, the signals must have the same duration. The final correlation values are normalized to the interval [−1, 1]. The greater the value to one, the better the correlation between the pair of channels considered, and negative values indicate the presence of phase difference correlation. In addition to the correlation between different channels, the decorrelation time is also calculated. The decoupling time indicates the time interval before the first transition from zero of the autocorrelation signal of each channel. The point at which the autocorrelation first resets to zero is the requested latency and is calculated for each available channel separately.

## 5. Long Short-Term Memory (LSTM) Crisis Prediction

**Figure 3:**
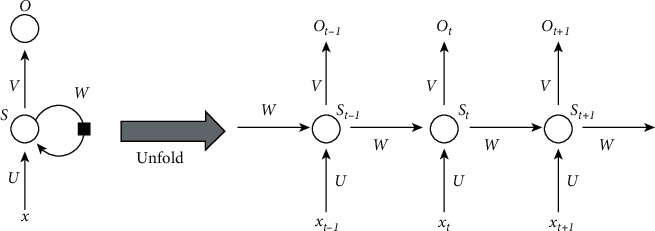
Architecture of a recurrent neural network (RNN) [18].

The architecture of the LSTM model is an evolution of recurrent neural networks (RNNs) and has been proposed to improve adaptability to sequential training data. RNNs were the first class of neural networks designed specifically for the analysis of sequential data and time series, and have found applications in the analysis of medical signals such as EEG. The peculiarity of this datum is that each value is a natural continuation of the previous one and depends on it. Patterns of time change also often appear, which in order to discover a network must be able to respond to current input given information that preceded it even long ago. RNNs are designed to manage such serial connections by allowing previous input values to influence the current response of the network, essentially implementing a type of memory as shown in Figure 3. Three tables with weights U, W, and V are used and applied for the input data for this purpose to the shared data in the hidden layer of the next state and to the network output, respectively. The weights are distributed among the different states based on the depth of the grid.

While theoretically long-range RNNs can be adapted to signals such as EEG, in practice they have been shown to be unable to detect long-range dependencies effectively. The reason is that the weight values in the *U*, *W*, and *V* tables are common to all time states supported by the depth of the grid, so it is very likely that some parameter will become unstable, causing the slope of the cost function rise or fall abruptly, which leads to a violation of the learning process and the impossibility of successfully adapting the network to the data due to the high complexity of the load calculation functions. This problem is commonly known as the gradient problem and is mainly related to back propagation in time [18].

### 5.1. Operation of LSTM Networks

**Figure 4:**
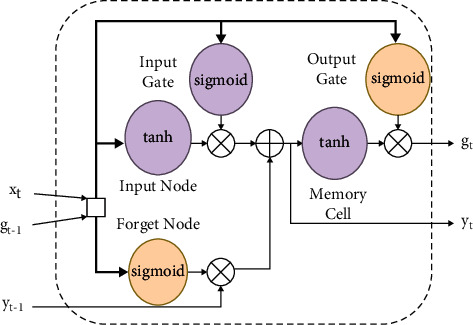
Interconnected memory cells of an LSTM network [20].

The previous difficulty of the internal architecture in an RNN was overcome with the long short-term memory model (LSTM) [19]. Although memory cells are wired in the same way as RNNs (Figure 3), LSTMs have a more complex architecture within them, allowing them to better manage information over long periods of time without requiring much effort to train them. LSTM is a chain-like structure constructed by replicating blocks of NN. The memory cell stores the information and runs the chain. In addition, the gates control whether information can eventually be added to or blocked in the memory cell. It is necessary to first create a vector for the cell state using the tanh function, then sort the information from *g*_*t*−1_ to *x*_*t*_, and multiply by the previously created vector to obtain the new output. LSTM functions are defined as in equations (2)–(7) as shown in Figure 4.

(2) $$\begin{array}{c} i_{t-g}=\phi \left(\omega_{i}\left[g_{t-1},x_{t}\right]\right)+a_{i}, \end{array}$$

(3) $$\begin{array}{c} f_{t-g}=\phi \left(\omega_{f}\left[g_{t-1},x_{t}\right]\right)+a_{f}, \end{array}$$

(4) $$\begin{array}{c} o_{t-g}=\phi \left(\omega_{o}\left[g.,x.\right]\right)+a_{0}, \end{array}$$

(5) $$\begin{array}{c} \bar{y_{t}}=\tanh\left(\omega_{c}\left[g_{t-1},x_{t}\right]\right)+a_{f}, \end{array}$$

(6) $$\begin{array}{c} y_{t}=f_{g}\cdot y_{t-1}+i_{g}\cdot \;\bar{y_{t}}, \end{array}$$

(7) $$\begin{array}{c} g_{t}=o_{g}\cdot \;\tanh\left(y_{t}\right). \end{array}$$

Logistic sigmoid function is represented by *ϕ*, hyperbolic tangent function is represented by tanh, and· denotes multiplication function. At time *t*, *i*_*g*_ denotes input gate, *f*_*g*_ denotes forget gate, *o*_*g*_ denotes output gate, and *g*_*t*_ denotes hidden state. *ω*_*i*_, *ω*_*f*_ and *ω*_*o*_ denote the weights of input, forget, and output gate, respectively, while *a*_*i*_, *a*_*f*_ and *a*_*o*_ denote their respective biases.

Therefore, by being able to independently install and configure three gateways for each memory cell, the LSTM network may be much more suitable for analyzing time-based data such as EEG recordings. Furthermore, an LSTM network can be composed of several hidden layers, in which the cache state output of one layer of memory cells is used as input for the next layer of memory cells, forming deeper and more complex architectures. However, the complexity of such a network grows rapidly, resulting in millions of training parameters for a network with multiple layers and a large number of memory cells in each layer. With networks this deep, there are two problems to solve:

1. Computational costs, which are enormous, and even arrays of computing units are required to train such networks.
2. Even if funds are available to cover the computational cost, a very large set of big data must be available for training to be efficient and not to overfit on the training data (overfitting).

For this reason, although advances in computers now provide a lot of computing power at a relatively low cost, the design of an LSTM network must be done very carefully to achieve the desired result with a minimum number of parameters possible, and the models should be generalizable and useful.

### 5.2. LSTM Network Architecture

**Figure 5:**
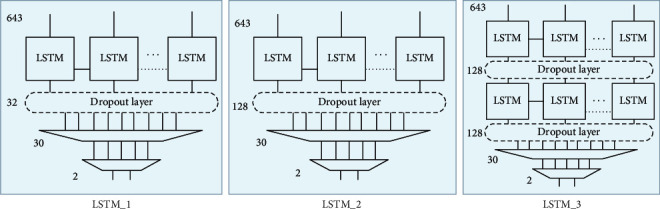
The proposed LSTM network architectures for crisis prediction.

Therefore, this section provides a preliminary analysis that considers three different LSTM network architectures, consisting of a simple circuit with multiple memory cells per layer and more complex networks with greater depth and number of elements. These networks will be tested with a small subset of EEG records from three randomly selected patients in the CHM-MIT database to assess classification accuracy between subcritical and intra-critical EEG segments and select the optimal architecture. The first architecture, LSTM_1, is the simplest approach with a grid consisting of a hidden level with 32 memory cells. In the second architecture, LSTM_2, the number of network memory cells is increased to 128 while maintaining a single layer design. In the third project, LSTM_3, the depth of the network is increased, one more hidden level is introduced, but the number of memory cells in each level remains constant: 128. Three architectures are shown in Figure 5. Figure 5 also shows intermediate levels of neuronal deletion (dropout). The use of dropout rates has been proposed as a method to solve the problem of training data overfitting in order to make LSTM networks more robust and generalizable to new samples. In practice, its purpose is to randomly reset part of each level's output in such a way as to remove a percentage of network information and make it difficult to learn very specific patterns that would be useless when evaluated with other data. The usefulness of this level in the present study of crisis forecasting is assessed below, since the size of LSTM networks is relatively small, and their contribution can be very limited. In all three cases, the output of the last layer of LSTM networks is complemented by two additional layers consisting of fully interconnected neural networks. The first takes the input of the LSTM network output and creates a 30-component output using the activation function of the rectified linear unit, ReLU [21]. The ReLU function is described by the equation:

(8) $$\begin{array}{c} f\left(x\right)=\text{max}\left(0,x\right), \end{array}$$

where *x* is the input. In practice, the function returns a value of zero for each negative input value, while for positive values, triggering occurs on a 45° dial ramp.

The ReLU function is preferred because it has been shown to perform better on deep machine learning network training problems and for this reason it is recognized as the most popular activation function [22, 23]. Finally, a second fully interconnected network produces a binary classification effect by dividing the EEG segments into qualitative or intercritical segments using the softmax function. The softmax function returns normalized values in the range [0, 1] for each network class. The class with the highest value is considered probably the most correct and is chosen to classify the corresponding EEG segment.

**Figure 6:**
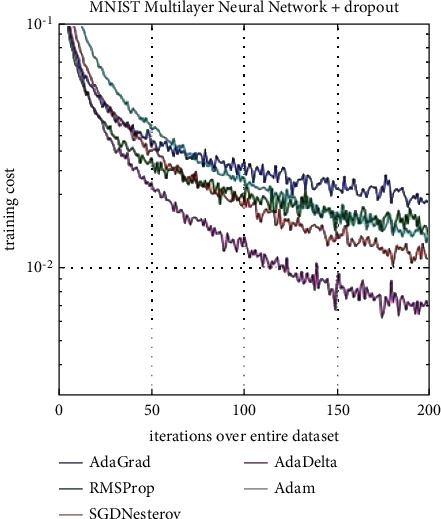
Computational cost of the Adam algorithm compared to corresponding optimization algorithms [24].

The cost function to train the algorithm uses a logarithmic cross-entropy function, and Adam's algorithm (adaptive momentum estimation) [24] was chosen as the optimization algorithm, using standard values of internal parameters (i.e., learning rate = 0.001, beta_1 = 0.9, beta_2 = 0.999, epsilon = 1*e* – 08, and decay = 0). As shown in Figure 6, the advantages of Adam's algorithm are lower computational costs and relatively faster convergence, which is a key factor for deep learning applications where network parameters can be very large and can be trained on large data sets. For this reason, although Adam's algorithm is a relatively recent implementation, it is almost installed on all deep learning network design platforms (e.g., TensorFlow, Keras, Torch, Caffe) as the recommended optimization algorithm.

Finally, due to the complexity of networks, the training process is carried out in smaller subsets of the total number of training samples, called batches, to limit the memory requirements of the system. In addition, in this way a smoother convergence is achieved when training the LSTM network. In each batch, pseudorandom training data subsets are selected based on a parameter value (e.g., for batch = 10, 10 samples) through an iterative process until all data samples have been used of training available. The process is then repeated for a predetermined number of iterations, called epochs. For the present study, both parameters (batch and epoch) are initialized to 10. The basic parameters of the network are shown in Table 1.

All LSTM network models for the needs of this work were implemented using libraries from the Keras package (version 2.0.9) [25] together with the TensorFlow environment [26]. Programming was done in Python 3.6.

## 6. Evaluation Results—CHB-MIT Database

The proposed seizure prediction methodology is evaluated using records from the CHB-MIT database and four different scoring windows at 15, 30, 60, and 120 minutes before seizure onset. The LSTM network is separately trained and evaluated for crisis prediction on each case from the CHB-MIT database. As in the preliminary analysis, the impact of choosing a different length of the LSTM network input sequence on the prediction accuracy is evaluated. The imbalance between the available sections in the two classes is resolved by dividing the intermediate sections into smaller subgroups of the same size as the classified class, and the results presented show the average of the measurements across all subgroups [27–30].

For a more complete evaluation, results are presented both for the case of segment-based evaluation and for the ability to predict events as events (event-based evaluation). To calculate the model performance, the following sampling parameters are defined as follows:

- True Positives (TP): The number of eligible partitions that are correctly classified as eligible.
- True Negatives (TN): The number of mesocritical regions that are correctly classified as mesocritical.
- False Positives (FP): The number of relevant sections misclassified as mesocritical.
- False Negatives (FN): The number of intermediate classes that are incorrectly classified as qualifiers.

For an evaluation based on the EEG components, from the above values, the sensitivity and specificity of the model are calculated as follows:

(9) $$\begin{array}{c} \text{Sensitivity}=\frac{TP}{TP+FN},\\ \text{Specificity}=\frac{TN}{TN+FP}. \end{array}$$

To assess evidence-based prognosis, each seizure is considered an independent event and sensitivity is defined as the percentage of successfully predicted seizures relative to the total number of seizures for each of 24 CHB-MIT-based cases. For a statement to be considered successful, at least one of its qualifying sections must be scored by the assessor as qualifying. Fact-based scoring also uses the false prediction rate (FPR), which indicates the number of false predictions per EEG.

### 6.1. Comparative Analysis

Table 1 presents a comparison of the proposed methodology with the international literature. The comparison focuses on studies that use the same database (e.g., the CHB-MIT Scalp EEG database). The proposed LSTM model provided better crisis prediction results than all previous methodologies that were previously evaluated with the same data set and using a similar qualification period. The exported features and the classification model used in each study are also presented in Table 2. Studies that did not use the classification algorithm but applied seizure prediction rules are marked with a “−.” With the exception of the graph-theoretic features, the other features extracted in the present analysis have previously been used successfully to predict seizures [31]. However, if previous studies did not use a large number of functions, according to the results of this evaluation, their combination provides a significant advantage, since the exported function space contains more and more essential information.

## 7. Conclusion

This work is dedicated to the development of artificial intelligence methods to improve the treatment of patients with epilepsy or Parkinson's disease, the two most common neurological conditions. In the treatment of Parkinson's disease, the affected patients with Parkinson's disease can assess the prognostic risk factors, and the symptoms are evaluated to predict rapid progression in the early stages after diagnosis. EEG seizure prediction, the superiority of LSTMs over CNNs has recently been reported in several applications related to EEG analysis. The presented seizure prediction system introduces deep learning algorithms into EEG score analysis. This proposed work long short-term memory (LSTM) network model is mainly implemented for the identification and classification of qualitative patterns in the EEG of patients. Compared to simpler classification models, as well as rule-based methodologies that rely on dynamic EEG changes, the proposed LSTM network demonstrates significantly higher overall seizure prediction accuracy.

Our future work, to enhance the work with optimization scheme based on artificial intelligence methods for accurate detection of patients with epilepsy or Parkinson's disease with less time consumption.

## Figures and Tables

**Table 1:** Basic parameters of the network.

Hidden state size LSTM	32 to 128
LSTM (layer number) levels	1 to 2
Fully connected layers	2 (ReLU, softmax)
Batch size	10
Epoch number	10
Sequence length	1 to 50
Learning rate	0.001
Number of classes	Procritic, mesocritical

**Table 2:** Comparison of the proposed crisis forecasting methodology with previous studies.

Study	Database	No. ofpatients	Number ofseizures	Total durationof EEG (hours)	Exported features	Classifier	Sens (%)	Spec (%)	FPR (*h*−1)	Qualifyingtime (min)
[24]	CHB-MIT	21	60	10.80	Phase synchronization	SVM	82.47^*∗*^	82.75	—	5
[25]	CHB-MIT	17	75	645	Absolute/relative energy distribution	SVM	98.66	—	0.045	55
[26]	CHB-MIT	10	30	60	Number of zero passages, similarity/dissimilarity coefficient	—	77.05	—	0.16	55
[27]	CHB-MIT	3	15	272	Number of zero passages, similarity/dissimilarity coefficient	—	83.86	—	0.164	35
	Private data	17	65	285	Number of zero passages, similarity/dissimilarity coefficient	—	91.25	—	0.06	35

[28]	CHB-MIT	13	120	434.4	Fourier transform	—	83.33	—	0.393	85
	Private data	3	15	148.1	Fourier transform	—	77.74	—	0.483	85

[29]	CHB-MIT	13	65	311.1	Spectrum images with STFT transform	CNN	81.20	—	0.14	5
[30]	CHB-MIT	15	15	70.4	Discrete transformation with wavelets	CNN	83.36	—	0.146	15
	Private data	12	14	24.20	Discrete transformation with wavelets	CNN	93.33	—	0.127	15

[31]	CHB-MIT	24	174	982.7	Eigenvalues of covariate tables	LDA	81.07	60.05	0.46	65
							87.06	50.03	0.3	95
							89.07	36.01	0.37	125

CNN-LSTM	CHB-MIT	24	180	978.2	Statistical values, number of zero passages,distinct transform. With wavelet, powerdistribution, and correlation between channels	LSTM	100/99.29^*∗*^	99.23	0.12	10
							100/99.37^*∗*^	99.67	0.07	25
							100/99.62^*∗*^	99.77	0.04	55
							100/99.81^*∗*^	99.85	0.03	115

SVM = support vector machine, CNN = convolutional neural network, LDA = linear discriminant analysis, Sens = sensitivity, Spec = specificity, FPR = false prediction rate per recording time. ^*∗*^Based on specified assessment field.

## Data Availability

The data sets used and/or analyzed during the current study are available from the corresponding author on reasonable request.
